# Vasculitis presenting as carpal tunnel syndrome: a case report

**DOI:** 10.1186/s13256-023-03801-8

**Published:** 2023-03-06

**Authors:** Mohammad Rahbar, Neda Dolatkhah

**Affiliations:** Physical Medicine and Rehabilitation Research Center, Tabriz University of Medical Sciences, Tabriz, Iran

**Keywords:** Carpal tunnel syndrome, Vasculitic neuropathy, Electrodiagnosis, Rehabilitation

## Abstract

**Background:**

Carpal tunnel syndrome is the most common focal mononeuropathy which presents with pain in the wrist and hand, paresthesia, loss of sensation in the distribution of the median nerve, and in more severe cases, weakness and atrophy of the thenar muscles. Meanwhile, carpal tunnel syndrome can present as an initial manifestation of underlying systemic vasculitis disorder and result in severe physical disabilities.

**Case presentation:**

A 27-year-old Iranian man was referred to our electrodiagnosis center with a clinical diagnosis of carpal tunnel syndrome in April 2020. Surgical intervention had been taken into account for him because of unsuccessful conservative therapies. On admission, thenar eminence was reduced. Electrodiagnostic findings were not compatible with median nerve entrapment at the wrist. All sensory modalities in the distribution of the right median nerve were decreased. Additionally, a mild increase in erythrocyte sedimentation rate was noted in laboratory tests. Because of the high vasculitis suspicion, we recommended the nerve biopsy and/or starting a high-dose corticosteroid. However, the surgery release was performed. After 6 months, the patient was referred for progressive weakness and numbness in the upper and lower limbs. After documentation of vasculitis neuropathy by biopsy, a diagnosis of non-systemic vasculitic neuropathy was confirmed. A rehabilitation program started immediately. Rehabilitation led to gradual improvement and recovery of function and muscle strength, and no complications remained, except mild leg paralysis.

**Conclusions:**

Physicians should be suspicious of the median nerve vasculitis mononeuropathy in a patient with carpal tunnel syndrome-like symptoms. Median nerve vasculitis mononeuropathy as an initial presenting feature of vasculitis neuropathy can further result in severe physical impairments and disabilities.

## Introduction

Carpal tunnel syndrome (CTS) is the entrapment and traction of the median nerve at the level of the wrist transverse carpal ligament and is the most prevalent upper extremity neuropathy, with a higher prevalence in the female population [1, 2]. The prevalence of CTS symptoms is 7.8 percent in the working population [3]. The characteristic presentation of CTS includes numbness, with or without “pins-and-needles” pain in the median nerve distribution area, including the palmar side of the first, second, and third fingers, and the radial half of the fourth finger, which often worsens at night or in the early morning [4]. Sometimes, patients’ description of symptoms is not completely consistent with median nerve involvement, such as symptoms perceived in all the fingers, the whole hand, forearm, arm, and shoulder, which also do not associate with the disease severity [5].

Conventionally, the diagnosis of CTS is clinically based. However, the diagnostic worth of several clinical examinations is controversial in the medical literature [6–8]. Furthermore, some patients with clinically diagnosed CTS have underlying rheumatologic, neurological, or musculoskeletal disorders such as peripheral polyneuropathy, cervical radiculopathy, plexopathy, proximal median mono-neuropathy, and misleading indicators in trauma that may describe their symptoms [9].

Vasculitis consists of a group of disorders, defined as the blood vessel wall destruction as a result of the infiltration of inflammatory cells [10]. Involvement of the peripheral nervous system is a usual complication of systemic vasculitis [11]. Although the typical form of peripheral nervous system involvement is mononeuritis multiplex (35–65%), it can present as focal axonal mononeuritis [10, 12]. Because of the step-by-step, progressive form of involvement, it is not unlikely to see mononeuritis at the initial stage of the disorder. The median nerve, in the area just proximal to the elbow, is one of the most involved nerves in this situation. This is important because the patient may be primarily supposed to have acute CTS [11].

As a result of these diagnostic problems, including uncharacteristic clinical features, suspicion of several diagnoses, ruling out a particular diagnosis, or establishing a clinical suspicion, a patient with probable CTS is referred for electrodiagnostic evaluation (EDX) [13, 14]. EDX may also be applied to assess the severity and prognosis, pre- and postoperative assessment and preparation of other management approaches, medico-legal and compensation matters, and baseline and screening destinations [15, 16].

EDX has become common in the CTS diagnosis since the 1950s, with a sensitivity of 78–95% and specificity of 80–95% [17–19]. The test measures showed median nerve physiological delays through reduced electrical conduction speed caused by compression, demonstrating the inflammation and axon loss degree [20]. This is valuable when the clinical findings are unclear [21, 22].

The application of EDX has some inadequacies. It is an invasive method with a long examination time and a high cost. Even for EDX-confirmed median neuropathy cases, different presentation is possible because of underlying probable systemic disease [23]. EDX abnormal findings do not predict the CTS severity. However, in many cases, when EDX is used together with clinical history and physical examination, the diagnosis can be done with a reasonable level of confidence [24].

Therefore, matching and comparing the EDX findings and CTS symptoms is very critical for physicians in clinical practice. In this way, suspicion should be focused on another existing pathology in the disparity between symptoms and EDX findings. In this article, we report a vasculitic neuropathy case in a 27-year-old man, and the symptoms of unilateral median nerve neuropathy in the initial stages of the disorder.

## Case presentation

A 27-year-old man from Iran was referred to the physical medicine and rehabilitation outpatient clinic at Tabriz University of Medical Sciences on 23 April 2020. He had no significant medical, family, or psychosocial history. First, he noticed pain and weakness in his right hand when he woke up on the morning of 20 March 2020 (1 month previous). Then, thenar atrophy gradually developed. Thenar atrophy and numbness of the hand did not improve with conservative treatments such as nonsteroidal anti-inflammatory drugs, physical methods (such as ultrasound therapy (US), low-level laser therapy), wrist splinting in the neutral position, and home exercises. He was diagnosed with CTS according to the clinical history and physical examination. Therefore, surgical intervention was considered for him. He was referred to our EDX center for redocumentation before surgical release.

He had no history of any injection at this region, and no evidence of systemic disease. On physical examination, he had right-hand thenar atrophy and numbness. About 2 weeks after the onset of the symptoms, a nerve conduction study (NCS) showed unobtainable sensory nerve action potential (SNAP) in median innervated fingers, and poorly evoked compound muscle action potential (CMAP) in abductor pollicis brevis (APB) muscle. NCS in other nerves of the right upper and left limb was normal. Needle electromyography (EMG) study of right thenar muscles revealed a reduced recruitment pattern with severe acute neurogenic changes.

On admission, an impaired function of the thumb (lack of ability to abduct and oppose the thumb, and decreased grip strength decrease of the affected hand) and lateral thenar muscle atrophy was evident. But the point to consider was that all sensory modalities had been reduced in the median nerve distribution and thenar eminence. Given that the thenar eminence is supplied by the median nerve palmar cutaneous branch, which branches off proximal to the carpal tunnel, the decreased sensation over the thenar eminence is not a common finding in CTS. So, we suspected a median nerve lesion proximal to the carpal tunnel and performed further NCS, and searched for other EDX explanations.

We noticed that the SNAP of the right palmar cutaneous branch of the median nerve was unobtainable. Additionally, the median nerve compound nerve action potential (CNAP) study in the forearm, while stimulating this nerve at the wrist and recording at the antecubital fossa, was unobtainable. By moving the nerve stimulation point toward the proximal, CNAP was still unobtainable up the junction of the distal one-third and proximal two-thirds at the forearm. Proximal to this junction, the nerve stimulation showed normal CNAP. The median nerve CNAP on the other side was normal. Needle examinations of median innervated right forearm muscles were normal.

These findings made us suspect “pseudo-CTS” (median nerve neuropathy at the lower forearm). This disorder is due to nerve entrapment or injury at locations other than the carpal tunnel, usually, the lower forearm, but shows similar signs and symptoms. By localizing the possible site of the median neuropathy by EDX, some potential causes of the median neuropathy at this region were compressive or sharp trauma to the forearm, a mass or ganglion cyst compressing the nerve, and vasculitis. The clinical exam was unremarkable for any penetrating injury or mass. Considering no history of any injection, and no evidence of systemic disease, we requested laboratory studies.

Surveys showed a mild increase in erythrocyte sedimentation rate (ESR) to 35 mm/h. The full blood count and other serologic markers, including antineutrophil cytoplasmic antibodies (ANCA), antinuclear antibodies, paraneoplastic antibodies, and anti-Ro/La antibodies, the immunologic markers for syphilis, hepatitis B and C, human immunodeficiency virus (HIV), and serum and urine protein electrophoresis were unremarkable. Routine urine analysis was normal.

Accordingly, we diagnosed median nerve vasculitic neuropathy and recommended neuropathy site exploration and/or starting a high dose of prednisolone therapy. Unfortunately, the attending physician and the patient did not accept these impressions and recommendations, and surgical intervention was performed.

After 6 months of surgery, the patient was referred to our center again. Now, he had “progressive weakness and numbness” in his upper and lower limbs and was not able to walk independently. He used an axillary crutch. The matter was that, over the 6 months after surgery, the patient had seen several attending physicians and finally had been hospitalized for diagnostic workups and treatment. The nerve biopsy sample had exhibited a reasonably severe injury of myelinated nerve fibers, in addition to the epineural arteriolar walls surrounding endoneurial microvessels and perineurium inflammatory infiltrations.

After the surveys, “nonsystemic vasculitis neuropathy (NSVN)” was diagnosed for the patient. The patient was referred after intravenous steroid pulse therapy and relative improvement of weakness and pain, for managing the remaining disabilities and impairments.

In the examinations, the skin and cranial nerves were normal. We failed to detect any evidence of systemic lesion. The deep tendon reflexes (DTRs) were absent, and all sensory modalities had decreased. Muscle atrophy was evident in the lower limbs, and to a lesser extent in the upper limbs. Motor clinical assessment revealed asymmetrically diminished motor strength (measured by British Medical Research Council muscle strength grading [25]) in the four limbs (3–5/5 in upper limbs, and 2–3/5 in lower limbs). NCS data revealed asymmetric axonal peripheral polyneuropathy, compatible with mononeuritis multiplex.

A rehabilitation program, which involved daily 1-h physiotherapy (PT) sessions for 3 days a week, started immediately.

Rehabilitation led to gradual improvement and recovery of function and muscle strength, and after 18 weeks of treatment, no complications remained except mild leg paralysis.

## Discussion

Here, we reported a 27-year-old man with unilateral hand thenar atrophy and numbness, later diagnosed as NSVN. The diagnosis of the patient was delayed due to the suspicion of CTS, which was followed by progressive motor weakness and paresthesia in his upper and lower limbs. This reminds us that occasionally uncommon neurological symptoms may show the onset of NSVN. On the other hand, a fraction of clinically suspicious patients with CTS may have other neurological diagnoses.

This case underscores the need for the EDX physician to be keenly aware of the various presentations of neurological and musculoskeletal disorders, and to perform a comprehensive neurological, musculoskeletal, and physical examination when necessary. Inquiries into work and recreational activities in addition to the laboratory tests may be useful. Patient management and referring physician satisfaction are optimized by these additional evaluations. Meanwhile, various other electrophysiologic, neurologic, and vascular disorders are also diagnosed.

NSVN is usually considered in the differential diagnosis of localized neuropathy, limited to the small arterioles of peripheral nerves, and probably muscles, without systemic involvement [26]. It is an uncommon disorder with necrotizing inflammation, causing peripheral nerve ischemic injuries. However, except for mild-to-moderate ESR elevation, the other indices of systemic inflammation are commonly absent. The EDX feature is a predominantly axonal nerve damage pattern. NSVN classification criteria are not consistent, and diagnostic criteria for vasculitic neuropathy are not partially identical because of different pathologic definitions of vasculitic neuropathy [27]. Patients present with stepwise progressive painful asymmetric multifocal distal predominant neuropathy, with acute or subacute onset. Sometimes early diagnosis might be challenging since the NSVN sequence is slow and can manifest with mononeuropathy, mononeuropathy multiplex, or symmetric polyneuropathy [28].

If mononeuropathy is seen in uncommon entrapment or compression region, the initial presentation of vasculitis neuropathy should be suspected. High clinical suspicion and early diagnosis can prevent other neuromuscular disorders and debilities due to peripheral and/or central neuropathy in the case.

EDX helps disclose typical vasculitic neuropathy findings, comprising acute or subacute axonal loss in sensory and motor nerve fibers, usually in an irregular, multifocal feature. However, when slower conduction or block at common entrapment locations is the only finding (such as in wrist median neuropathy), physicians should consider other reasons for possible compression neuropathies, such as diabetes and nonvasculitic rheumatoid arthritis (RA) [29].

Laboratory study should consist of a complete blood count (CBC), metabolic assessments (electrolytes, blood urea nitrogen (BUN), creatinine (Cr), and fasting blood glucose), ESR, c-reactive protein (CRP), in addition to antinuclear antibody (ANA), rheumatoid factor (RF), antineutrophil cytoplasmic antibodies (ANCA), hepatitis B and C, and cryoglobulins [30]. Serum complement measurements are requested in suspected mixed cryoglobulinemia or systemic lupus syndromes (SLE). Serum protein electrophoresis, measurement of concentrations of extractable nuclear antigen and angiotensin-converting enzyme (ACE), and HIV infection assessment are also required [31].

When vasculitis is doubted in such patients, the peripheral nerve biopsy is very valuable. Furthermore, due to the long-term treatment necessity with possible toxic medication, the vasculitis diagnosis generally requires histological confirmation [31]. In this study, biopsy specimens showed severe myelinated nerve fiber injuries due to inflammatory infiltrations.

NSVN treatment is principally immunosuppressive therapy, started as soon as possible. The standard immunosuppressive therapy is a combination of corticosteroids (prednisone) and cyclophosphamide (CYC), methotrexate (MTX), or azathioprine for 7 to 12 months. In corticosteroid monotherapy, a follow-up is required and when the signs of neuropathy progression are seen, CYC, MTX, or azathioprine must be added. The NSVN prognosis is better than other systemic vasculitides [32]. Frequently, neurological deficits resolve over time, and the disease is remitted for years without relapsing.

We guess that numerous patients with the clinical suspicion of CTS referred to community-based EDX settings may have other musculoskeletal and neurologic disorders instead. We further hypothesize that patients with EDX findings consistent with CTS have more diverse clinical presentations than patients whose analyses are negative for CTS. Identification of patients with positive and negative clinical presentations may allow the referring medical doctor to select more precisely the patients who would benefit most from EDX studies.

## Conclusion

This case emphasizes the importance of suspecting NSVN in acute-onset symptoms identical to severe CTS. Median nerve EDX can help with differential diagnosis in such patients. Additionally, this case showed a suspected rare disorder—when there are atypical or treatment-refractory symptoms, it is very important for physicians in clinical practice to take note of these.

## Data Availability

Data sharing is not applicable to this article as no datasets were generated or analyzed during the current study.
